# When Herbivores Eat Predators: Predatory Insects Effectively Avoid Incidental Ingestion by Mammalian Herbivores

**DOI:** 10.1371/journal.pone.0056748

**Published:** 2013-02-12

**Authors:** Matan Ben-Ari, Moshe Inbar

**Affiliations:** Department of Evolutionary and Environmental Biology, University of Haifa, Haifa, Israel; CNRS, Université de Bourgogne, France

## Abstract

The direct trophic links between mammalian herbivores and plant-dwelling insects have been practically ignored. Insects are ubiquitous on plants consumed by mammalian herbivores and are thus likely to face the danger of being incidentally ingested by a grazing mammal. A few studies have shown that some herbivorous hemipterans are able to avoid this peril by dropping to the ground upon detecting the heat and humidity on the mammal's breath. We hypothesized that if this risk affects the entire plant-dwelling insect community, other insects that share this habitat are expected to develop similar escape mechanisms. We assessed the ability of three species (adults and larvae) of coccinellid beetles, important aphid predators, to avoid incidental ingestion. Both larvae and adults were able to avoid incidental ingestion effectively by goats by dropping to the ground, demonstrating the importance of this behavior in grazed habitats. Remarkably, all adult beetles escaped by dropping off the plant and none used their functional wings to fly away. In controlled laboratory experiments, we found that human breath caused 60–80% of the beetles to drop. The most important component of mammalian herbivore breath in inducing adult beetles and larvae to drop was the combination of heat and humidity. The fact that the mechanism of dropping in response to mammalian breath developed in distinct insect orders and disparate life stages accentuates the importance of the direct influence of mammalian herbivores on plant-dwelling insects. This direct interaction should be given its due place when discussing trophic interactions.

## Introduction

Mammalian herbivores (MH) affect the life history and populations of small arthropods in various ways [1]. Many studies have examined the effects of grazing mammals on arthropod assemblages (e.g. [2], [3]) and mechanisms underlying these effects. One such mechanism is a density mediated indirect effect: MH alter the arthropods' food availability by changing plant community assemblage [4]. Another such influence is a trait mediated indirect effect: grazing changes the growth architecture [5], chemistry [6] or distribution pattern [7] of plants that serve as food or shelter for plant-dwelling insects (PDI).

While indirect effects of MH on PDI communities have been studied extensively (e.g. [8], [9], [10]), direct interactions between these two groups have been greatly overlooked. Direct interactions between species are fundamentally important for understanding food web structure and energy flows in communities. MH can directly affect PDI in two ways - trampling (e.g. [11], [12]) or direct feeding. Tscharntke [13] suggested that MH may directly influence PDI communities by incidentally ingesting insects, specifically those that live inside plant tissues, such as gall-makers and tissue-burrowing larvae, along with the plant during grazing.

MH feed on a plethora of plant species and plant parts, which are also the habitat and food source of many PDI, and the two groups are bound to interact. Surprisingly, the documentation of incidental ingestion of PDI by MH is scarce, but examples exist for its effect on insect eggs [14], larvae [15] and adults [16]. Nevertheless, despite the high probability for this interaction, when trophic links in a food web are discussed, direct incidental ingestion of insects is often disregarded. Since most authors deal only with intentional feeding, direct trophic links between creatures with such a disparity of body size have been considered unlikely because of the difficulty in prey handling and location [17].

Until recently, research on incidental ingestion of insects by mammalian herbivores was limited mostly to sessile insects or sessile life stages of motile species, e.g., burrowing larvae [15] or gall-inhabiting insects [18]. The reason for the significant effect large herbivores had on these endophagous insects was their inability to move when the large herbivore approached. Thus, the only behavioral mechanism available to them for avoiding incidental ingestion by MH was preferential selection of an egg-laying or galling site location that is out of reach of the herbivore's mouth (e.g. [19], [14]). Mobile insects, however, face the same threat: recent experimental evidence has begun to emerge as to the direct effect of incidental ingestion by MH on motile insects and the behavioral mechanisms these insects have developed to avoid such ingestion. One such behavioral mechanism is dropping off the plant in response to the presence of a MH.

Different predators of insects may bring about a dropping response (e.g. [20], [21], [22], but it also helps herbivorous insects avoid being incidentally consumed by MH. Gish et al. [23], [24] have shown that two species of aphids (Hemiptera: Aphididae) are able to detect an approaching MH by sensing the heat and humidity of its breath, which combined are a reliable cue for the presence of an herbivore. A large proportion of the aphid colony then opts to drop from the plant in order to avoid almost certain destruction through ingestion by the large herbivore.

It is clear that aphids are not the only motile PDI threatened by mammalian herbivores. The PDI community comprises a large variety of insects of different orders. Any insect perching on an edible plant might incidentally fall prey to the approaching herbivore. If indeed the direct effect of MH is pervasive, we expect to see other PDI that have evolved mechanisms for detecting grazing herbivores and effectively escaping incidental ingestion. If MH affect populations of PDI of other trophic levels, such as predators and parasitoids, they may influence the community structure and food webs that include the associated insect herbivores.

In order to effectively determine whether this phenomenon is widespread we need to explore the response to the threat of incidental ingestion by insects that are phylogenetially remote and physiologically different from aphids. Such insects should be distinct from aphids in the several important criteria: phylogeny, trophic level and feeding mode, host specificity (as opposed to oligophagous aphids) and mobility.

Predators of aphids, which habitually share the aphids' habitat, are prime candidates for investigating the ubiquity of the ability of PDI to avoid incidental ingestion. One such group of predators which has been studied extensively is coccinellid beetles (Coleoptera: Coccinellidae), which differ from aphids in all the prerequisite criteria but share their habitat. Both adults and larvae of many species in this family are voracious predators of aphids, sometimes even used as important agents of biological control [25]. Adults and larvae are not strictly confined to the plant's surface but often remain in or around the aphid colony [26] and are thus susceptible to incidental ingestion by MH. The only thing linking these two disparate groups is the common danger they are exposed to.

Using three species of aphidophagous coccinellids, we examined three main questions: (1) Will these beetles effectively avoid being eaten along with a plant when it is grazed upon by MH? (2) What cues are used by coccinellid beetles to detect MH and avoid incidental ingestion? (3) Will this response manifest in both adults (winged) and larvae (wingless)?

## Materials and Methods

### Beetles and plants

The three beetle species used in this research – *Coccinella septempunctata*, *Scymnus frontalis* and *Chilocorus bipustulatus* – are important aphid predators that have been used as biological control agents, targeting aphids and scale insects [27]. All species were collected in gardens around Haifa in northern Israel. No specific permissions were required for the collection of the study animals, since they were collected in public urban gardens and not in nature reserves. The locations were not privately owned. Furthermore, these beetles are not protected animals and require no permit for collection.

*C. septempunctata* adults and larvae were collected mainly from mallow plants (*Malva* sp.). *C. bipustulatus* adults and *S. frontalis* adults and larvae were collected from Spanish Broom bushes (*Spartium junceum*). The adults and larvae were reared individually in plastic Petri dishes with tissue paper as a perching spot for the beetle, and fed every two days on live 2^nd^–3^rd^ instar aphid nymphs of two species (*Acyrthosiphon pisum* and *Afis fabae*). The beetles were reared in constant conditions of 22°C and 60–65% humidity and a 16L:8D light regiment. Experiments took place in the laboratory under the same ambient conditions as the rearing, during the daytime. All insects were handled with soft featherweight tweezers to avoid physical damage.

### The ability of *C. septempunctata* to avoid incidental predation

To assess the beetles' ability to avoid incidental predation, we tested the proportion of beetles that dropped off the plant in response to an approaching herbivore. The experiment was conducted in a 2×2 m cage in the Kibbutz Hahotrim petting zoo. A group of 10–11 *C. septempunctata* larvae or 10 adults was placed on a 30 cm high potted alfalfa plant (16 cm diameter flower pot) (*Medicago* sp.) that was placed the middle of a cage. The beetles (larvae or adults separately) were allowed to settle for 15 min, and then a goat (*Capra hircus*), reared in the petting zoo, was allowed to enter the cage and feed on the plant for 20 seconds. The goat was then taken out of the cage and the number of intact individuals found on the ground was counted. The treatment was replicated three times for adults and for larvae. Individuals that walked or flew off the plant before the experiment began were excluded from the experiment.

### The response of coccinellids to cues related to mammalian presence

To study the response of adult beetles and larvae to different cues associated with a mammalian herbivore's presence, we conducted a series of controlled lab experiments.

Each individual (adult or larva) was placed on a separate two-week old broad-bean plant (*Vicia faba*) planted in a small plastic cup (diameter 7 cm, height 10 cm). Each plant was host to a colony of 10–20 *A. fabae* aphids as food source for the beetles. The individual was given time to settle (30 min for larvae and 15 min for adult beetles, as preliminary results indicated that by these times the beetles stopped moving and started feeding) and was then subjected to one of the experimental treatments. Larvae were used only in their 3^rd^–4^th^ instars to avoid lack of response by the larvae due to imminent pupation. Larvae and adults that showed signs of prolonged agitation or attempts to fly off the plant were excluded from the experiment. During the experiments, the experimenter's mouth and nose were covered with a surgical mask to avoid breathing on the beetles.

Each beetle/larva was subjected to all the seven experimental treatments and the control treatment, except for the larvae of *S. frontalis*, which were not subjected to the vibration and tactile stimulation treatments. The order of the different experimental treatments was randomized between species and developmental stages. Due to the nature of the breath simulation apparatus, which requires extended time periods to be calibrated to the exact heat and humidity, the order of the treatments was not randomized within a group, i.e. all individuals of a certain species underwent the treatments in the same order. After being subjected to a treatment, the beetles were removed from the plant and returned to their rearing Petri dishes, and were allowed to rest at least one hour before undergoing another treatment.

#### Heat and humidity components of breath:

First we tested whether beetles respond to the same cues as did their aphid prey, i.e. the heat and humidity of mammalian breath [23], [24]. Breath simulation treatments were performed using the mammalian breath apparatus as described by Gish et al. [23]. This apparatus allows us to produce a constant air flow (air stream velocity of 0.5 m/sec) at preordained temperature and humidity by bubbling filtered air through water at various temperatures. The four treatments performed by the apparatus were:

1. Control: air temperature 22°C, relative humidity 60–65% (similar to ambient conditions).
2. Heat: air temperature 35–36°C, relative humidity 60–65%.
3. Humidity: air temperature 22°C, relative humidity 80–90%.
4. Heat + Humidity: air temperature 35–36°C, relative humidity 80–90%.

The Heat treatment was provided via a specially adjusted version of the apparatus, whereby the air flow was inserted into a heated dry compartment and excess fluids were collected in a small vial in order to reduce the humidity of the air flow.

Each treatment was applied for 2 seconds with the muzzle of apparatus held 2 cm from the adult/larva. The muzzle was held next to the beetle when the latter was on a stem or a leaf positioned perpendicularly to the ground to ensure that a beetle letting go of the plant would indeed drop from it.

These treatments were also compared to a Human Breath treatment, which emulated the overall effect of an approaching mammal's muzzle: the beetles were lightly breathed upon by the senior author with an open mouth in a fashion equivalent to 0.5 m/s wind (also see [23]). The breathing was performed at a distance of 2–5 cm for a two-second interval.

#### Additional cues related to mammalian herbivore presence:

We also examined several additional cues that might be used by the beetles to detect approaching MH.

Carbon dioxide is present at elevated levels in the breath of mammals and could also be a reliable cue for herbivore presence. We tested the effects of CO_2_ on beetles. The experimental setup was as described above (air temperature of 22°C and relative humidity of 60–65%, similar to ambient conditions) with an air mixture containing 5% CO_2_, similar to the content of mammalian exhalation.

Two other effects that can be associated with herbivore feeding are the vibration it causes in the host plant [28] and the touch of the air and moving plant parts on the beetle's body. Since the effects of air stream movement are tested in the Control treatment described above, we tested for the effect of the tactile stimulation of touch.

In the Vibration treatment, the plant's base was vibrated for two seconds using a plastic rod attached to a small electrical motor with an extending flap. When activated, the flap repeatedly struck the rod, moving it and creating a constant vibration at a frequency of 12 movements per second and amplitude of 0.1 cm. The end of the rod was placed at the base of the plant, 1 cm above the ground.

In the Tactile Stimulation treatment, the individual was lightly touched on its elytra or abdomen (for the adults or larvae, respectively) with a fine hair three times in a period of 2 seconds.

### Statistical analysis

The initial sample sizes were 30 adults and 30 larvae of *C. septempunctata*; 17 adults and 30 larvae of *S. frontalis* and 23 adults of *C. bipustulatus*. Since some individuals were excluded in certain treatments the number of replicates varied. In *C. septempunctata* adults, the Heat treatment was compared for 26 individuals, and the vibration and tactile stimuli treatment were compared for 27 individuals. In *C. septempunctata* larvae, the heat, vibration and tactile stimuli treatments were compared for 24 individuals. In *C. bipustulatus* adults, the humidity treatment was compared for 22 individuals. In *S. frontalis* larvae, the human breath and heat treatments were compared for 26 individuals.

The overall effect of treatment, developmental stage and beetle species was appraised using a generalized linear mixed model (GLMM) with a binomial error distribution, due to the binary nature of the dependent variable. The developmental stage and air-flow treatment were included as fixed factors and the individual nested within species as a random factor. The breath treatments included the Control, the different heat and humidity comninations, Human Breath and the CO_2_ treatment.

The difference in proportions of falling beetles/larvae was compared between the different treatments using McNemar's test for comparing proportions in dependent samples. Since the data in every group were compared to five other treatments(e.g. the Control treatment was compared to the Heat, Humidity, Heat+Humidity, Human Breath and CO_2_ Treatments), the Bonferroni correction was applied and the significance level was set at α = 0.01. Due to the very low dropping rates of the vibration and tactile stimuli treatments, they were not compared statistically to avoid further reduction in significance level. Analysis was performed using SPSS PASW software, version 19.

## Results

### The ability of *C. septempunctata* to avoid incidental predation

Both larvae and adults of *C. septempunctata* were able to avoid incidental ingestion when the goat consumed the plant they were standing on. Even though the goat readily consumed the alfalfa plant in all experiments within 20 seconds, an average of 82.1±1% (STD) of the larvae and 91.3±1% (STD) of the adults were not harmed as they dropped to the ground. All the individuals that did not drop to the ground were consumed by the goat, as we did not find beetles on the remaining plant stems or on the goat itself. Remarkably, no adult beetle escaped the plant by flying away, but rather the fleeing beetles dropped to the ground.

### The response of coccinellids to cues related to mammalian presence

#### Heat and humidity components of breath:

**Figure 1:**
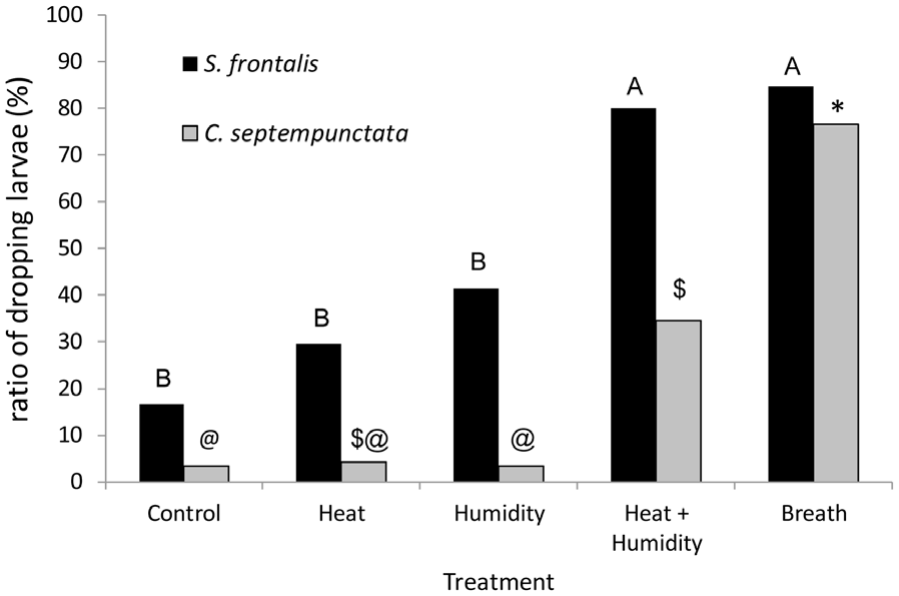
Dropping rates of beetle larvae in response to breath and its simulation. The actual dropping rates (not averages) of larvae of *Scymnus frontalis* and *Coccinella septempunctata* in response to the different airflow treatments. Each species was statistically analyzed separately (McNemar's test, α = 0.01). Columns marked with the same uppercase letter are not significantly different. Columns marked with the same symbol are not significantly different.

**Figure 2:**
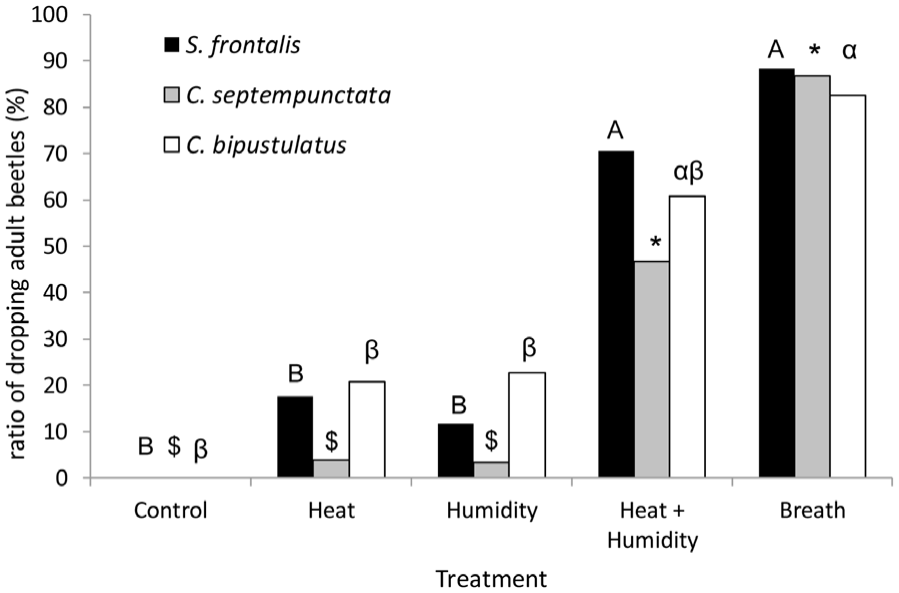
Dropping rates of adult beetles in response to breath and its simulation. The actual dropping rates (not averages) of adults of *Scymnus frontalis*, *Coccinella septempunctata* and *Chilocorus bipustulatus* in response to the different airflow treatments. Each species was statistically analyzed separately (McNemar's test, α = 0.01). Columns marked with the same uppercase letter are not significantly different. Columns marked with the same symbol are not significantly different. Columns marked with the same Greek letter are not significantly different.

In general, the three beetle species (adults and larvae) responded similarly to the different breath simulation treatments. The results of the GLMM showed a significant fit in both the model as a whole (F_2,747_ = 48.3, p<0.001) and in the air-flow treatment factor (F_1,747_ = 98.391, p<0.001) but not in the developmental stage factor (F_1,747_ = 2.779, p = 0.09). When the different treatments were compared within a species, in all of the test groups the human breath treatment elicited the highest dropping rates (76–88%), followed by the heat + humidity treatment (34–80%). Even though the heat + humidity treatment produced lower dropping rates than the Breath treatment, the differences were significant only in *C. septempunctata* larvae; both treatments differed significantly from the Control treatment (Figs. 1 and 2). No beetle reacted to any of the treatments by flying off the plant, and the only responses were either remaining on or dropping off the plant.

In all three species, the control treatment produced no response in adults (Fig. 2). The adults and larvae were less responsive to the separate heat treatment and humidity treatment than they were to the combined treatment, and on the whole were irresponsive to the control treatment (Fig. 1, 2). In the GLMM analysis, the interaction between the developmental stage and treatment was significant (F_1,747_ = 4.92, p = 0.027), though not high, considering the large sample size. And indeed, the response patterns of the different species and life stages were similar but not uniform. *C. septempunctata* were two times more inclined to drop due to the human breath treatment than due to the heat + humidity treatment, while other species did not show such a significant difference. In addition, larvae of *S. frontalis* were somewhat responsive to the Heat treatment and the humidity treatment while larvae of *C. septempunctata* showed almost no response to these separate treatments (Fig. 1).

Coccinellid larvae attach themselves to the plant surface with an adhesive structure in the posterior end of their abdomen called the anal disc [29]. We observed that larvae of *C. septempunctata*, which were on the upper, axial side of the broad bean leaf, detached the anal disc from the surface when breathed upon, curled their bodies and rolled off the leaf surface (data not shown).

#### Additional cues related to mammalian herbivore presence:

No beetle species (both larvae and adults) showed a pronounced response to the vibration and tactile stimulation treatments, with only a single individual at the most dropping in each treatment (Table 1). The CO_2_ treatment also induced almost no dropping behavior. Only adults of one species, *C. bipustulatus*, responded in proportions that approached significance (McNemar's test, p = 0.03) to this treatment compared with the control treatment.

**Table 1:** The actual (not average) ratios (%) of dropping individuals of the different beetle species and developmental stages in response to the Tactile Stimulation, Vibration and CO_2_ treatments.

Species	N	Control	Tactile	Vibration	CO_2_
*C. septempunctata* Larvae	24	3.33	4.17	4.17	7.14
*C. septempunctata* Adults	27	0	3.70	0	3.70
*S. frontalis* Larvae	25	16.67	Not tested	Not Tested	20
*S. frontalis* Adults	17	0	11.76	5.88	11.76
*C. bipustulatus* Adults	23	0	0	4.17	26.09

Due to the very low dropping rates in the tactile and vibration treatments, only in the CO_2_ treatment each group was statistically compared to the respective group in the control treatment (McNemar's test, α = 0.01). No result was statistically significant.

## Discussion

Coccinellid beetles evade incidental ingestion by MH very effectively. Both adults and larvae of the three species examined were able to detect the presence of an herbivore by sensing its breath and dropped to the ground to avoid being consumed. In the feeding experiments, almost all *C. septempunctata* (larvae and adults) dropped to the ground and were not eaten by the approaching goat.

Dropping in response to mammalian breath is apparently a widespread phenomenon. Aphids and coccinellids are very disparate in their phylogenies, physiological traits and life histories [30], [27]. Nevertheless, these distinct insect groups are both members of the PDI community and their shared habitat exposes them to the common risk of incidental ingestion by MH. Thus, both groups have developed a similar mechanism for dealing with this common threat. While it is possible that not all coccinellid species share this behavior or the specific cue it relies on, the three non-congeneric species in this research are indicative of the pervasiveness of this behavioral mechanism in the coccinellid family.

The fact that coccinellids have adapted to sensing mammalian breath and evolved a behavioral mechanism for avoiding incidental ingestion points to the centrality of the direct threat that grazing MH impose on these beetles and the PDI community in general. It is reasonable to assume that other PDI cohabiting on plants with coccinellids are faced with this threat and have developed similar mechanisms. While detailed information as to the cost dropping to the ground incurs is still lacking, works done on aphids suggest that this cost is substantial. Young, vulnerable aphids are more reluctant to leave the plant than the larger adults [28] and adult aphids have developed mechanisms to reduce the chance of reaching the ground after dropping from a leaf [31]. Such costs on the ground may also affect beetles and, even more pronouncedly, their larvae, which are less mobile. The decision to drop, despite the possible costs, shows the extent of the possible selection pressure MH exert on PDI.

An ecological community consists of various organisms with varying physiological and behavioral traits. When such disparate members of the same community evolve a behavioral mechanism convergently for avoiding a common threat, this means that this threat affects their entire habitat. Thus, the main factors that would elicit a community-wide response in PDI are those that affect the entire plant. For example, most PDI living in habitats with periodical fires have evolved traits important for avoiding fires and recolonizing the damaged habitat [32]. MH cause immediate and extensive damage to plants and plant patches, thus radically changing the habitat of smaller organisms residing on these plants. PDI living in grazed habitats or on grazed plants are likely to develop traits allowing them to detect, escape or avoid this threat.

Our results suggest that incidental ingestion by MH has a pronounced direct effect which is not restricted to herbivorous insects. Hence, MH may alter community structure and trophic cascades in PDI communities in complex, sometimes contrasting ways. MH reduce plant material and affect its quality [8]; they can affect herbivorous insect populations by incidentally consuming them and they may reduce predation pressure on herbivorous insects by feeding on predators, such as coccinellids. Such an effect on several trophic levels simultaneously might liken MH to keystone predators, which prey on and affect the populations of many different groups in their habitat both directly and indirectly [33].

All of the beetle species examined dropped in response to breath, as well as to hot and humid air flow, much like aphids. The air movement by itself, as represented by the control treatment, was insufficient to dislodge the beetles and induce significant dropping rates. Therefore, this dropping reaction does not stem from the inability of beetles to hold on to the plant but rather is an active behavioral mechanism brought on by a specific cue.

The most important cue for inducing the dropping response was the combination of heat and humidity. Other signals related to herbivore presence, i.e. the vibration and the tactile stimuli treatments, elicited the dropping response in neither adults nor larvae, similarly to aphids [23], [24]. This result serves to enhance the role of a puff of hot and humid air as the most reliable cue for the presence of an herbivore in the field. This cue is effective and reliable for several reasons: Heat and humidity are specific to MH breath and do not change suddenly due to environmental factors and seasonal changes; they are a ubiquitous character of mammalian breath, unrelated to the herbivore's diet or size; the puff of hot and humid air occurs only when herbivore feeding is imminent, thus reducing erroneous dropping; and finally, unlike visual cues, heat and humidity can be perceived during night hours. Other possible natural enemies of coccinellid beetles, such as birds, are not likely to be the source of such a cue as most of them do not rely on the sense of smell to locate prey, and do not sniff the plant prior to feeding.

It is possible that in the different groups of PDI facing the common threat of incidental ingestion, other mechanisms arose for accurately detecting mammalian presence, beyond heat and humidity. Adults of *C. bipustulatus* (nymphs were not tested) showed some response to the CO_2_ treatment which was higher (nearly significantly) than the Control treatment. Some arthropods, in particular blood-sucking parasites of mammals, have been shown to respond to and orient towards a CO_2_ source [34], [35]. Nevertheless, we do not know why CO_2_ has had no evident effect on aphids and other coccinellids (Table 1; [23], [24]).

Dropping off the plant is sometimes used by coccinellid larvae as a defense mechanism against intraguild predation by lacewings and other coccinellid beetles [36], [37]. This type of response is instar-specific, as smaller instar larvae are more easily captured and eaten and therefore drop to the ground more readily when an arthropod predator attempts to catch them [38]. In our research, adults and larvae of the same species dropped in similar proportions, illustrating the severity of the threat by a mammalian herbivore, regardless of the beetles' developmental stage. Moreover, the cue inducing the dropping response in this research, i.e. hot and humid air, could be caused by an approaching mammal but not by invertebrate natural enemies of the beetles. Other signals that might be indicative of an intraguild insect predator, such as plant vibration or tactile contact, did not induce coccinellids to drop.

Unlike their larvae, adult coccinellids have the ability to fly as another method of escaping incidental ingestion by MH. Flying away and dropping off a plant may be seen as competing strategies for avoiding a potential predator within the same species of an insect [39]. Surprisingly, adult beetles responded to breath or its simulation by dropping and not by flying away. Even the vigorous shaking that accompanied the actual feeding on the plant by the large herbivore did not cause a single beetle to fly away.

When flying insects, such as moths [40], lacewings and mantises [41], detect an approaching bat, they engage in evasive movements. While a distant, searching bat induces the moth to simply fly in the opposite direction, when faced with an imminent threat from a bat closing in on it, the moth moves downwards in a series of maneuvers [42]. Among these are passive dives whereby the insect falls downward without flapping its wings in order to steer out of the bat's course. In much the same way, dropping from the plant may allow the beetles to quickly disengage and move away from the herbivore's muzzle without pausing to unfold their wings. Indeed, most of them (91%) were able to avoid incidental ingestion by dropping to the ground.

The fact that other PDI have developed this kind of behavioral mechanism will affect the way we view the role of incidental ingestion in shaping insect communities. Since direct links between MH and PDI have been virtually ignored, food web descriptions tend to either group insect and vertebrate herbivores together [43] or discuss only one of the groups separately, as parts of disparate sub-webs (e.g. [44]). Few authors (e.g. [13]) integrated grazing mammals and PDI of different guilds into the same description of trophic links. Integrating the two separate food-web descriptions could provide a fuller, truer picture of the trophic interactions in grazed habitats.

The three species examined in this study have all been used as biological control agents and have therefore received considerable attention, most notably *C. septempunctata*. When studying the beetles' various traits, there is a disparity between results gathered in the field and in those obtained in lab arenas (e.g. [45]). While many environmental factors are dissimilar between field observations and laboratory experiments, our study suggests that interaction with mammalian herbivores might also affect the dispersal and population of coccinellids, and thus their efficacy as biological control agents.

## Conclusion

The direct interaction between large MH and PDI is not a phenomenon limited in scale. We believe that the existence of a behavioural response to the danger of incidental ingestion by MH in an insect group which is phylogenetically and trophically distinct from aphids suggests that MH have a direct effect on PDI in general and exert substantial selection pressure on them. The importance of this direct link should therefore be taken into account when considering the various trophic interactions in natural and grazed habitats and be added to the complexity of food web links.
